# Supplementary material for: Lactiplantibacillus plantarum A72, a Strain with Antioxidant Properties, Obtained through ARTP Mutagenesis, Affects Caenorhabditis elegans Anti-Aging
Source: Foods. 2024 Mar 19;13(6):924. doi: 10.3390/foods13060924 (PMC10969348; doi:10.3390/foods13060924)
Supplement: Supplementary file 1 [file foods-13-00924-s001.zip › foods-2896285-supplementary.pdf]

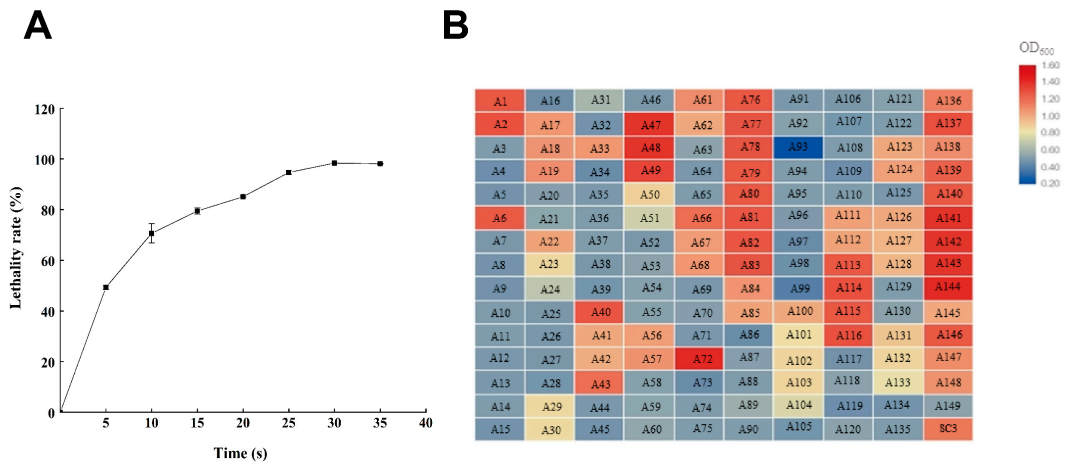

Figure S1 Results of ARTP mutagenesis selection of *Lpb. plantarum* SC3 as the parent strain. (A) ARTP mutagenesis lethality curve. (B) The growth results of 149 mutant strains and *Lpb. plantarum* SC3 in MRS broth with 1.5 mM H_2O_2. The color of the heatmap indicates the OD_{600} value of the strains after 20 h in MRS broth with 1.5 mM H_2O_2, and the redder the color, the better the growth.

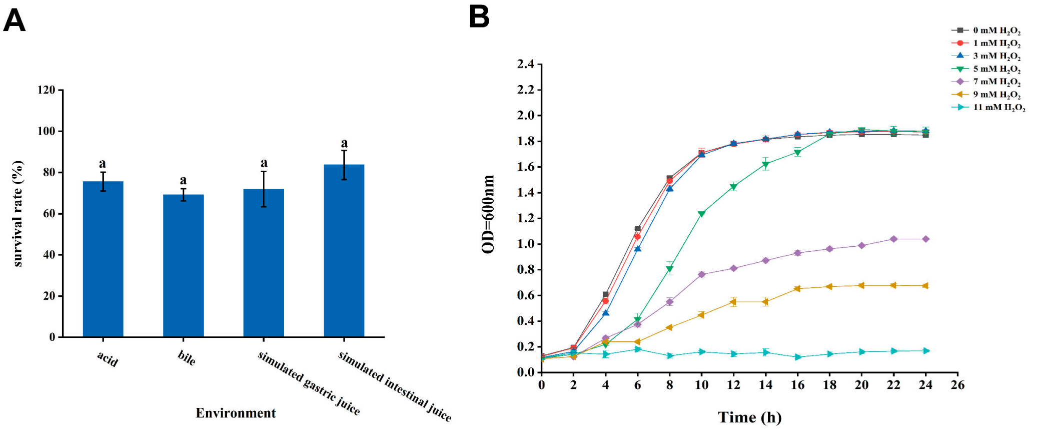

Figure S2 Beneficence and growth curve of *Lpb. plantarum* A72. (A) Survival of *Lpb. plantarum* A72 cultured for 4 h in MRS broth at pH = 3.0, MRS broth with a bile salt concentration of 0.3% (w/v), artificial gastric fluid and artificial intestinal fluid. (B) Growth curves of *Lpb. plantarum* A72 in different concentrations of H_2O_2 MRS broth. [a] *Lpb. plantarum* A72 was tolerant to acid, bile salts, artificial intestinal fluid and gastric fluid environments and the four tolerances were not significant.
